# High-temperature-resistant silicon-polymer hybrid modulator operating at up to 200 Gbit s^−1^ for energy-efficient datacentres and harsh-environment applications

**DOI:** 10.1038/s41467-020-18005-7

**Published:** 2020-08-24

**Authors:** Guo-Wei Lu, Jianxun Hong, Feng Qiu, Andrew M. Spring, Tsubasa Kashino, Juro Oshima, Masa-aki Ozawa, Hideyuki Nawata, Shiyoshi Yokoyama

**Affiliations:** 1 Institute for Materials Chemistry and Engineering, Kyushu University, 6-1 Kasuga-koen Kasuga, Fukuoka, 816-8580 Japan; 2 The University of Aizu, Fukushima, 965-8580 Japan; 3 Department of Molecular and Material Science, Kyushu University, 6-1 Kasuga-koen Kasuga, Fukuoka, 816-8580 Japan; 4 Nissan Chemical Corporation, Funabashi, 274-0069 Japan; 5 Tokai University, Kanagawa, 259-1292 Japan

**Keywords:** Fibre optics and optical communications, Optoelectronic devices and components, Polymers, Silicon photonics

## Abstract

To reduce the ever-increasing energy consumption in datacenters, one of the effective approaches is to increase the ambient temperature, thus lowering the energy consumed in the cooling systems. However, this entails more stringent requirements for the reliability and durability of the optoelectronic components. Herein, we fabricate and demonstrate silicon-polymer hybrid modulators which support ultra-fast single-lane data rates up to 200 gigabits per second, and meanwhile feature excellent reliability with an exceptional signal fidelity retained at extremely-high ambient temperatures up to 110 °C and even after long-term exposure to high temperatures. This is achieved by taking advantage of the high electro-optic (EO) activities (in-device *n*^3^*r*_33_ = 1021 pm V^−1^), low dielectric constant, low propagation loss (*α*, 0.22 dB mm^−1^), and ultra-high glass transition temperature (*T*_g_, 172 °C) of the developed side-chain EO polymers. The presented modulator simultaneously fulfils the requirements of bandwidth, EO efficiency, and thermal stability for EO modulators. It could provide ultra-fast and reliable interconnects for energy-hungry and harsh-environment applications such as datacentres, 5G/B5G, autonomous driving, and aviation systems, effectively addressing the energy consumption issue for the next-generation optical communication.

## Abstract (web summary)

Information and communication datacentres require a large amount of energy for their cooling systems, which could be decreased by working at higher temperatures. Here, the authors introduce a silicon-polymer hybrid modulator that maintains high data rates for long periods at high temperatures that could be used under such conditions, to reduce energy consumption.

## Introduction

Recently, the traffic in data communications has seen explosive growth due to the emergence of bandwidth-hungry applications, such as high-definition streaming media, 5G/B5G, cloud-based service delivery and Internet-of-Things. The rapid growth in datacentre’s count and power density is leading to a dramatic increase in demand for energy. It is predicted that by 2025, datacentres will account for 20% of worldwide electricity consumption^1,2^. The cooling systems alone may account for up to 40% of the energy demands of a datacentre^3^. Therefore, to increase energy efficiency in datacentres, one effective solution is to raise the operating temperature, thus lowering the energy consumption in the cooling systems. Four per cent operating costs could be saved from cooling for every 1 °C increase in operating temperature^4^. However, this puts forward higher requirements for the reliability and long-term stability of the components in the datacentres, especially transceivers, at high ambient temperatures. As one of the key components in the transceiver, electro-optic (EO) modulators have been implemented on several photonic platforms, such as silicon^5–8^, indium phosphide (InP)^9–11^, organic^12–16^, lithium niobate^17–22^ and plasmonics^23–29^, each with their own advantages, such as enabling the integration with complementary metal-oxide-semiconductor (CMOS) electronics, low drive voltages, ultra-high bandwidths up to the terahertz regime^22–24^ and small footprints. Considering the ever-increasing energy consumption and emerging applications in harsh environments, apart from the aforementioned aspects, the reliability of the EO modulator at high ambient temperatures is another important aspect to be investigated. Recently, several directly modulated quantum-dot^30^ and distributed feedback (DFB)^31,32^ lasers have been successfully demonstrated operating at an elevated temperature of up to 80 °C with data modulations at 25 and 53 Gbaud, respectively. On the other hand, external EO modulators incorporating organic EO materials, such as silicon-polymer hybrid (SPH), silicon-organic hybrid (SOH) and plasmonic-organic hybrid (POH) modulators, have shown outstanding thermal reliability owing to the intrinsic high glass transition temperatures (*T*_g_) of the deployed organic EO materials^25,29,33–36^. We have recently reported a thermally stable EO polymer modulator demonstrating stable performance of its static parameter (half-wave voltage, *V*_π_) at elevated ambient temperatures up to 105 °C for 2000 h^33^. Thereafter, we have further optimized the shape, the nonlinearity and the *T*_g_ of the EO polymer using advanced molecular engineering, and improved the electronic and photonic circuits, especially the electrode design.

Herein, we demonstrate a high-temperature-resistant ultra-high-speed SPH modulator, exhibiting an extremely high bearable ambient temperature of up to 110 °C with maintained high-speed signal fidelity, meanwhile ensuring high EO activities (in-device EO figure of merit: *n*^3^*r*_33_ = 1021 pm V^−1^ at 1.55 μm, where *n* is the refractive index and *r*_33_ the in-device EO coefficient), and achieving up to 200 Gbit s^−1^ aggregate data rates, specifically, up to 120 Gbit s^−1^ with on–off keying (OOK) and up to 200 Gbit s^−1^ with four-level pulse-amplitude modulation (PAM4). Furthermore, the excellent signal fidelity at 100 Gbit s^−1^ is also experimentally confirmed even after long-term (100 h) exposure to 90 °C, thus validating the long-term thermal stability of the modulator. No energy-consuming temperature stabilization unit is deployed in the system. The simultaneous fulfilments of high thermal stability and ultra-high-speed signalling make technological inroads into energy-efficient and high-throughput datacentres. In addition, it can also be used to implement reliable and ultra-fast interconnects in harsh-environment applications.

## Results

### SPH modulator

**Fig. 1:**
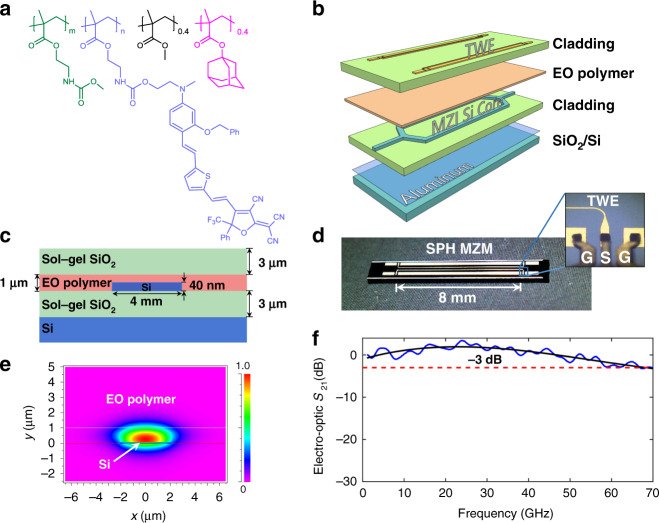
Silicon-polymer hybrid (SPH) modulators. **a** Molecular structure of the synthesized side-chain EO polymer. **b** Schematic diagram of the fabricated SPH modulator by layers. **c** Cross-sectional view of the phase shifter section of the SPH modulator. **d** Top view photograph of a fabricated SPH modulator chip. Inset: close-up micrograph of the electrode section of the device with an RF probe. The ground contacts penetrate to the bottom electrode of the device. **e** Numerically simulated optical field distribution in the cross-section of the modulator. **f** Measured EO bandwidth (*S*_21_ parameter) of the SPH modulator. The measured 3-dB bandwidth of the SPH modulator is around 68 GHz. The solid black line represents the fitted curve.

To fabricate the SPH modulator, we prepare side-chain EO polymers with an ultra-high *T*_g_ of up to 172 °C, which are synthesized according to a modified procedure based on ref. ^37^. As shown in Fig. 1a, a chromophore with high-molecular hyper-polarizability is attached to the polymer backbone with a high loading density to realize the second-order nonlinear effect, that is, Pockels effect, and provide a high EO coefficient, thus enabling efficient EO modulations. Meanwhile, the bulky adamantyl unit branched in the acrylate polymer leads to a high *T*_g_. Since the thermal relaxation of acentric chromophore order in the poled film is negligible as long as the storage temperature is well below the *T*_g_, the EO polymers with high *T*_g_ could offer good thermal stability. In the polymer synthesis, the loading densities of the chromophore and adamantyl can be easily adjusted, resulting in EO polymers with different *T*_g_ and EO efficiencies. A *T*_g_ of EO polymers up to 172 °C is obtained with a loading density of adamantyl at 34 wt%, while the loading density of chromophore of ~37 wt% leads to an effective in-device EO figure of merit (*n*^3^*r*_33_) of around 1021 pm V^−1^ at a wavelength of 1.55 μm (Supplementary Note 2). Moreover, to further enhance the thermal stability of the EO polymer, dialysis purification is performed to remove polymers with low molecular weights. In contrast, the conventional EO polymers are prepared by mixing EO chromophores in the polymer hosts. However, such guest–host polymers could not sufficiently meet the demands for industrial applications due to incomplete thermal reversibility with molecular disordering at elevated temperatures. An in-device material property comparison of our synthesized EO polymer to the state-of-the-art organic EO materials is provided in Supplementary Table 1.

The synthesized EO polymer is then used to fabricate the SPH modulator, which is fabricated in a travelling-wave Mach–Zehnder interferometer (MZI) configuration, consisting of ultra-thin silicon core, side-chain EO polymer, sol–gel SiO_2_ claddings and electrodes. Figure 1b illustrates the schematic structure of the fabricated SPH layer by layer. The MZI waveguide is patterned on a 40-nm-thick silicon layer. The strip waveguide consists of two Y-junctions, which split and combine the optical power, and two 8-mm-long arms with a 200 μm spacing. As illustrated in the schematic of the cross-section of the hybrid waveguide (Fig. 1c), a 1-µm-thick EO polymer layer is sandwiched between two cladding layers prepared from the sol–gel SiO_2_ resin. A top view photograph of the fabricated SPH modulator is presented in Fig. 1d with a close-up micrograph of the electrodes of the device. On the top of the device, microwave strip-line gold (Au) electrodes with a length of 8 mm are deposited on the MZI arms. To achieve a large EO bandwidth, the electrodes are operated in a travelling-wave manner and optimized for impedance matching (Supplementary Note 3). Here, the thickness and width of the electrodes are designed to be 3 and 16 μm, respectively. More importantly, in contrast to crystalline and amorphous material modulators, the intrinsic low dielectric constant of the EO polymer results in comparable refractive indices of the microwave and light signals, thus leading to negligible velocity mismatching between them to enable high-speed and broadband operations of the device. According to the measured frequency response of the fabricated SPH modulator shown in Fig. 1f, although the analyser has a limited frequency bandwidth (see “Methods”), a 3-dB bandwidth of 68 GHz is observed, while the 6-dB bandwidth of the device is presumed to be over 70 GHz.

To ensure a high EO efficiency, in addition to the optimization of the material’s EO coefficient, the light should be well confined between silicon and polymer layers. Figure 1e shows the calculated field distribution of the TM_0_ mode. Given that the refractive indices of the polymer and silicon are 1.67 and 3.48, respectively, according to the modal calculation (Supplementary Note 1), the confinement factor (*Γ*) defined by the ratio of the optical power in the EO polymer to the total power is 73.8% with a 4-µm-wide and 40-nm-thick ultra-thin silicon core, which is higher than conventional silicon on insulator (SOI) strip waveguides with typical geometrical dimensions, for example, *Γ* ≈15% with a 220 × 500 nm silicon core^38^. With such a large confinement factor in the modulator, the induced refractive-index change in the EO polymer cladding, which is proportional to the applied electrical field, results in a corresponding change of mode field distribution, and consequently accomplishes effective modulations of the guided light. The advantage of the intrinsic high EO coefficient of the polymer, together with the excellent confinement of light in the waveguide, enables a high EO efficiency, leading to a measured π-voltage–length product (*V*_π_⋅*L*) of 1.44 V⋅cm at a wavelength of 1.55 μm. In addition, in such a shallow silicon strip^39–41^, the fundamental optical mode near the sidewall occupies a small fraction of the total available optical power, thus leading to a propagation loss as low as 0.22 dB mm^−1^ (Supplementary Note 5).

### Up to 200 Gbit s^−1^ signalling

**Fig. 2:**
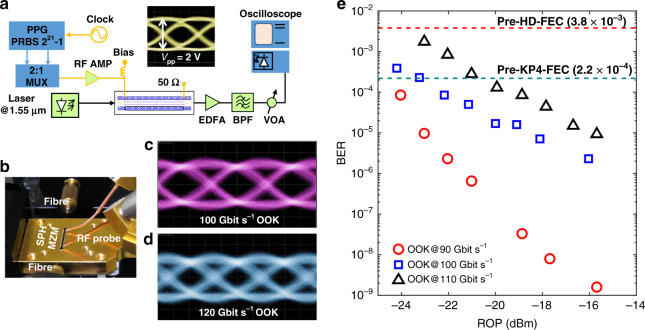
100 Gbit s^−1^ and beyond OOK signalling. **a** Experimental setup for generating OOK signals from our fabricated SPH modulator. PPG pulse-pattern generator, MUX electronic multiplexer, AMP amplifier, VOA variable optical attenuator, BPF optical band-pass filter, EDFA erbium-doped fibre amplifier. Inset shows the applied driving electrical 100 Gbit s^−1^ OOK signal with a peak-to-peak voltage of 2.0 V. **b** Photograph of the experimental setup for testing high-speed operation up to 120 Gbaud. **c**, **d** Measured optical eye diagrams of OOK signals at data rates of 100 Gbit s^−1^ (**c**) and 120 Gbit  s^−1^ (**d**). The corresponding *Q* factors are 7.3 and 5.4, respectively. **e** Measured BER curves as functions of the received optical power (ROP) for 90, 100 and 110 Gbit s^−1^ OOK signals. The pre-FEC BER thresholds for hard-decision (HD) and KP4 FECs^42^, that is, 3.8 × 10^−3^ and 2.2 × 10^−4^, respectively, are also plotted for reference.

As mentioned above, the optimizations of the electronic circuit, such as electrode dimensions, impedance matching, minimization of frequency-dependent radio frequency (RF) propagation loss and coupling loss, together with the intrinsic low dielectric constant of EO polymer, lead to a measured 3 dB bandwidth of around 68 GHz, showing the possibility for data operation beyond 100 Gbit s^−1^. The bandwidth expansion is expected by further optimizing the travelling-wave electrode design and fabrication. Using binary driving electronics (Fig. 2a, b), OOK signals at bit rates up to 120 Gbit s^−1^ are generated. The measured optical eye diagrams of 100 and 120 Gbit s^−1^ OOKs are shown in Fig. 2c and d, respectively, with clear eye openings. The corresponding bit-error rates (BERs) at operating rates of 90, 100 and 110 Gbit s^−1^ are presented in Fig. 2e when the received optical power (ROP) before the photo-detector is adjusted. No error floor is observed even for 110 Gbit s^−1^ OOK till BER of 10^−5^, which is well below the pre-KP4-FEC (forward error correction) BER threshold (2.2 × 10^−4^)^42^. As presumed in the bandwidth analysis, error-free operations are achieved at bit rates beyond 100 Gbit s^−1^, validating the high-speed operating capacity of the device.

**Fig. 3:**
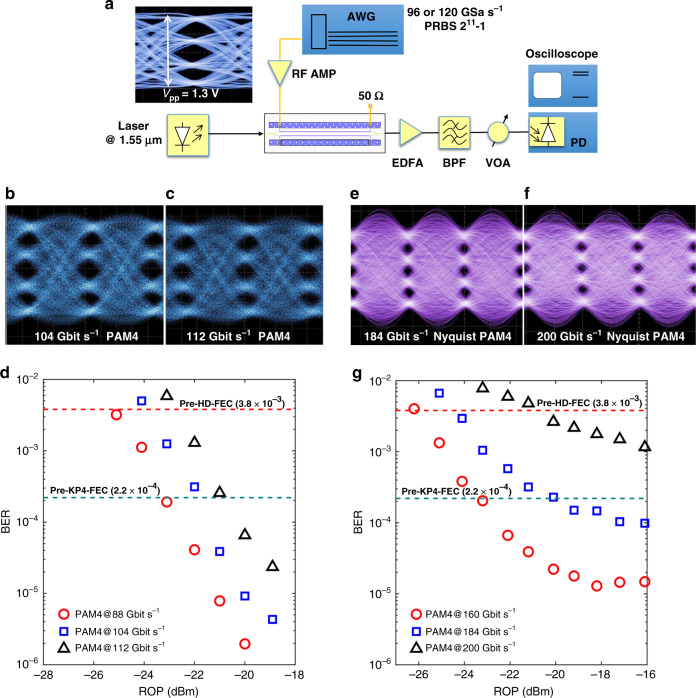
Up to 200 Gbit s^−1^ PAM4 signalling. **a** Experimental setup for generating PAM4 signals from our fabricated SPH modulator. AWG arbitrary waveform generator. Inset shows the driving electrical PAM4 signals with a peak-to-peak voltage of 1.3 V. **b**, **c** Measured optical eye diagrams of PAM4 signals at data rates of 104 Gbit s^−1^ (**b**) and 112 Gbit s^−1^ (**c**). **d** Measured BER curves as functions of the received optical power for 88, 104, and 112 Gbit s^−1^ PAM4 signals. **e**, **f** Measured optical eye diagrams of Nyquist PAM4 signals at data rates of 184 Gbit s^−1^ (**e**) and 200 Gbit s^−1^ (**f**). **g** Measured BER curves as functions of the received optical power for 160, 184, and 200 Gbit s^−1^ Nyquist PAM4 signals. The pre-FEC BER thresholds for hard-decision (HD) and KP4 FECs^42^, that is, 3.8 × 10^−3^ and 2.2 × 10^−4^, respectively, are plotted for reference.

Multilevel modulation is another effective method to increase the operating rate of the modulator. As shown in Fig. 3a, for PAM4 signalling, a four-level electrical signal from an arbitrary waveform generator (AWG), used as a digital-to-analog converter (DAC), at a sampling rate of 92 GSa s^−1^ is deployed to drive the SPH modulator. Optical PAM4 signals at symbol rates varying from 44 to 56 Gbaud are generated, achieving operating rates up to 112 Gbit s^−1^. Clear eye diagrams of the obtained 104 and 112 Gbit s^−1^ PAM4 signals are shown in Fig. 3b and c, respectively. The measured BER curves of synthesized 88, 104 and 112 Gbit s^−1^ as functions of ROPs are depicted in Fig. 3d. No error floors are observed up to BER of 1 × 10^−5^ for all the measured BER curves at bit rates up to 112 Gbit s^−1^. Instead of conventional non-return-to-zero pulse shape, Nyquist pulse shaping with a small roll-off factor (0.01) could be helpful to further boost the signalling speed of the device with enhanced spectral efficiency. By applying raised cosine filters over the driving electrical signals, through another DAC operated at 120 GSa s^−1^, Nyquist PAM4 signalling at 80, 92 and 100 Gbaud are realized by the SPH, achieving operating rates of 160, 184 and 200 Gbit s^−1^, respectively. The obtained eye diagrams at 184 and 200 Gbit s^−1^ are shown in Fig. 3e and f, respectively. According to the measured BER curves illustrated in Fig. 3g, the error rates below both the pre-HD-FEC (3.8 × 10^−3^)^42^ and pre-KP4-FEC BER thresholds are well achieved for both 160 and 184 Gbit s^−1^ Nyquist PAM4. On the other hand, for the synthesized 200 Gbit s^−1^ Nyquist PAM4, the error rates below the pre-HD-FEC threshold are also observed. The successful syntheses of signals beyond 100 Gbit s^−1^ (up to 200 Gbit s^−1^) in either OOK or PAM4 verify the ultra-fast operating capacity of the fabricated SPH modulator.

### High-speed operation at elevated temperatures

In addition to ultra-fast signalling, the deployment of a high-*T*_g_ EO polymer in the SPH modulator allows for high-temperature operation without failure, which is essential for improving the datacentres’ energy efficiency by raising the ambient temperature, and crucial for providing components suitable for harsh environments in 5G^43^, LiDAR, satellites and avionics applications. We have tested the long-term thermal reliability and stability of the device in accordance with Telcordia standards of high-temperature storage. It has been confirmed that the static EO characteristic (*V*_π_) of our EO polymer modulator converges to a constant long-term stable value over a long duration (2000 h) at elevated temperatures of 85 and 105 °C.

**Fig. 4:**
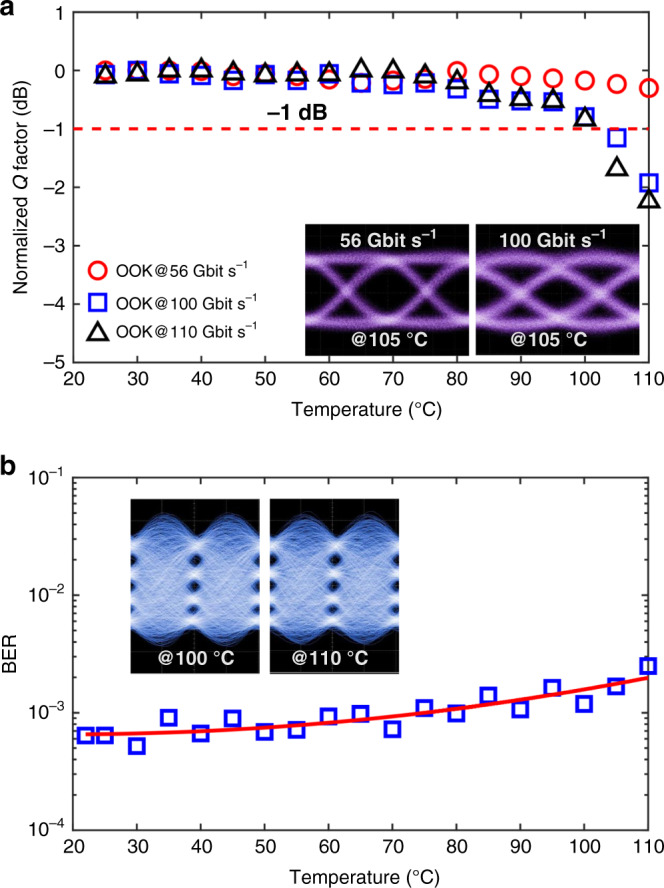
Up to 200 Gbit s^−1^ operating at elevated temperature. **a** Normalized *Q* factors (dB) of the synthesized OOK signals at 56, 100 and 110 Gbit s^−1^ as a function of the operating ambient temperature (25–110 °C). Inset: measured optical eye diagrams of 56 and 100 Gbit s^−1^ OOK signals at elevated temperature (105 °C). The unnormalized *Q* factors versus the operating ambient temperatures can be found in Supplementary Figure 4. **b** Measured BERs of 200 Gbit s^−1^ Nyquist PAM4 versus operating ambient temperatures (22–110 °C). The solid red solid line represents the fitted curve. Inset: measured optical eye diagrams of 200 Gbit s^−1^ Nyquist PAM4 at temperatures of 100 and 110 °C.

In order to further comprehensively assess the high-temperature reliability of SPH modulators, instead of monitoring static parameters of the device such as *V*_π_, optical insertion loss, and *S* parameters, it would be more effective and informative to measure dynamic properties like BER or *Q* factor of the signals synthesized from the device during or after the exposure to elevated ambient temperatures. In contrast to previous investigations, for the first time to our knowledge, the thermal reliability of the fabricated SPH modulator is assessed by investigating its high-speed performance when the operating ambient temperature is adjusted over an extraordinary wide range (25–110 °C). For the test, our modulator is first configured as a binary OOK transmitter by feeding binary driving electrical signals at bit rates of 56, 100 and 110 Gbit s^−1^. The corresponding *Q* factors of the synthesized OOK signals are measured and shown in Fig. 4a when the ambient temperature is adjusted over the aforementioned range. As shown in the inset of Fig. 4a, clear eye openings are observed at 56 and 100 Gbit s^−1^ even at extremely high temperatures up to 105 °C. When the ambient temperature is increased from 25 to 110 °C, 56 Gbit s^−1^ OOK shows <0.5 dB decrease in the measured *Q* factor. The measured *Q* factors of 100 and 110 Gbit s^−1^ OOK signals decrease slightly (~1 dB) when the ambient temperature is increased up to 100 °C. Although higher bit-rate signalling becomes more sensitive to ambient temperatures, the change of *Q* factor within the wide temperature range is <2.5 dB for both 100 and 110 Gbit s^−1^ signals. The measured *Q* factors over the wide temperature range are all >3.51, corresponding to the KP4 pre-FEC BER threshold. No abrupt *Q* factor degradations are observed even for OOKs operating at 100 Gbit s^−1^ and beyond. As presented in the next section, a negligible penalty is observed once the device is cooled down to room temperature even after long-term high-temperature (90 °C) exposure, which is in line with previous investigations^33^. In addition, the thermal reliability of the SPH modulator is also investigated when the device is operating at 200 Gbit s^−1^ with PAM4. The BERs of the synthesized 200 Gbit s^−1^ PAM4 are measured over the aforementioned ambient temperature range and plotted in Fig. 4b, only showing a slight increase of less than one order of magnitude. These results indicate the excellent thermal reliability of the SPH modulator at high temperatures, which is attributed to the stable ordering of the chromophore in the synthesized ultra-high-*T*_g_ EO polymer. More importantly, the high EO activities of the SPH modulator are well retained over a wide temperature range, which enables up to 200 Gbit s^−1^ high-speed operation even at high ambient temperatures up to 110 °C.

### High-speed operation after long-term high-temperature storage

**Fig. 5:**
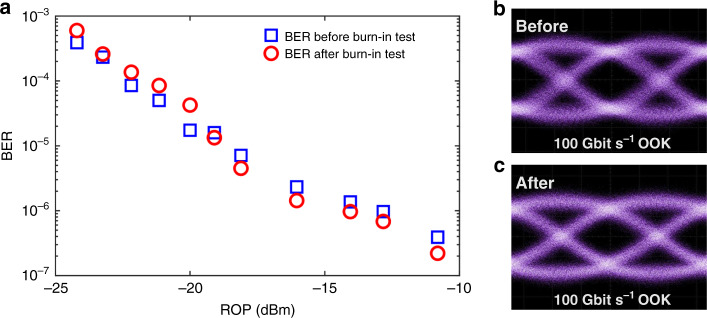
100 Gbit s^−1^ operating after long-term high-temperature storage. **a** Measured curves of BER versus the received optical power (ROP) of 100 Gbit s^−1^ OOK signals before and after the long-term (100 h) high-temperature (90 °C) exposure. **b**, **c** Measured 100 Gbit s^−1^ OOK optical eye diagrams before (**b**) and after (**c**) the high-temperature exposure.

As for the organic EO material integrated in SPH devices, de-poling (molecular disordering) usually occurs ~<30 °C below the *T*_g_^44,45^, implying that the upper temperature limit for stable operation of the side-chain EO polymer used in our SPH device (*T*_g_: 172 °C) is ~140 °C. To validate the high-speed functionality of the SPH modulator even after a burn-in high-temperature test, the BER performance of the generated 100 Gbit s^−1^ signals is evaluated after 100-h exposure to a high temperature (90 °C). For comparison, the measured curve of BER versus the ROP before the burn-in test is measured and plotted. As shown in Fig. 5a, error-free operations are confirmed for 100 Gbit s^−1^ signals before and after the burn-in test with BER up to 1 × 10^−5^, which is below the KP4 pre-FEC BER threshold. With respect to the BER curve before the burn-in test, negligible power penalty is observed even after the 100-h exposure to high temperature, which is consistent with the observed negligible degradation of *V*_π_ after a period of high-temperature storage^33^. Clear eye openings are observed in the measured eye diagrams in both cases, shown in Fig. 5b and c, respectively. The results indicate that the high EO activities could be maintained even after being stored at elevated temperatures for a long term, demonstrating an excellent long-term thermal stability of the fabricated broadband SPH modulator.

### Thermal reliability comparison

EO modulators are mainly formed on structures like micro-ring resonator (MRR), MZI or a combination of MRR and MZI. MRR exhibits a compact footprint, but shows a strong sensitivity to ambient temperature fluctuations, especially on a high thermo-optic (TO) coefficient substrate such as silicon. Most of the proposed approaches to overcome the thermal instability issue in MRR either require significant power consumption in the temperature stabilization unit^46,47^ or incorporate negative TO coefficient materials at the cost of increased complexity^48–50^. Through the active compensation approach, athermal silicon MRR modulators with a good thermal tolerance against 15 K^46^ or 7.5 °C^47^ thermal drift have been successfully demonstrated. In contrast, MZI and MRR-assisted MZI^51^ structures possess better thermal stability. These structures have been demonstrated on various photonic platforms, such as SOI, lithium niobate, InP, SOH, POH and SPH. Table 1 lists and compares some modulator metrics of our device with prior demonstrations, focusing on the operating speed and the operating temperature range when the devices are high-speed activated. Note that, for the POH^25,29^ and InP^10^ modulators, the high-temperature reliability is evaluated by monitoring the static properties of the devices at a single frequency such as *V*_π_, modulation index or efficiency, rather than evaluating the high-speed signal quality from the devices. To compare the EO activity of these devices across different photonic platforms, herein a voltage-bandwidth parameter is defined by the ratio of the measured 3 dB bandwidth over the half-wave voltage of the device, which is also known as bandwidth half-wave voltage ratio (BVR). A larger BVR indicates a high-speed operating ability with a low driving voltage, corresponding to higher EO activity and efficiency. As shown in Table 1, our device shows the highest EO activity (BVR: 38 GHz V^−1^). As another important figure of merit for EO modulators, loss-efficiency product is quantified by the multiplication of π-voltage–length product (*V*_π_ · *L*) and the propagation loss (*α*), that is, *α* · *V*_π_ · *L*. In comparison to other devices in Table 1, although the SPH modulator presented in this work has no clear advantage in terms of the footprint, it features the best loss-efficiency product (3.6 V · dB), indicating an excellent trade-off between optical loss and modulation efficiency. More importantly, it is the only one achieving up to 200 Gbit s^−1^ operations under an extraordinary wide temperature range (25–110 °C). The fabricated EO polymer modulator on a silicon substrate represents a new paradigm in EO modulator development for ensuring excellent high-temperature reliability and up to 200 Gbit s^−1^ ultra-high-speed operations. It simultaneously meets the requirements for both the operating speed and thermal reliability of optoelectronic components in future energy-efficient datacentres and harsh-environment applications.

**Table 1:** Comparison of several metrics for EO modulators.

Platform	Structure	EO bandwidth (GHz)	Propagation loss (*α*, dB mm^−1^)	BVR (GHz V^−1^)	Half-wave voltage (*V*_π_, V)	Footprint, diameter (Ø) or active length	Loss-efficiency product (V⋅dB)	Line rate (Gbit s^−1^)	Format	Operating temperature range or Δ*T*
LNOI^17^	MZI	15	0.3	1.7	9	2 mm	5.4	22	OOK	Δ*T* = 20 °C
SOI^51^	MRR-assisted MZI	N/A	N/A	N/A	N/A	0.796 mm	N/A	1	OOK	22.5–100 °C
SOI^46^	MRR	N/A	N/A	N/A	N/A	Ø: 0.01 mm	N/A	1	OOK	Δ*T* = 15 K
SOI^47^	MRR	21	2.9	30	0.7	Ø: 0.0048 mm	N/A	25	OOK	Δ*T* = 7.5 °C
SOI^8,a]^	MZI	N/A	0.05	N/A	N/A	0.04 mm^2^	N/A	20	OOK	25–125 °C
POH^25^	MZI	>70	375	>12.5	~12	0.016 mm	75	72	OOK	20–80 °C
								116	PAM4	
POH^29^	Straight slot	>65	~414	>1.45	44.8	0.029 mm	~538	40	BPSK	−85 °C
InP^10^	MZI	37	0.69	14.8	2.5	4 mm	6.9	43	OOK	−40 °C
SOH^34^	MZI	20	1.7	13.5	1.48	1.5 mm	3.74	40	OOK	−85 °C
SOH^35^	MZI	32	9.3	14.5	2.2	0.75 mm	15.81	128	PAM4	−80 °C
SPH^36^	MZI	60	0.22	30	2	8 mm	3.5	56	OOK	20–95 °C
								112	PAM4	
SPH	MZI	68	0.22	38	1.8	8 mm	3.2	110	OOK	25–110 °C
This work								200	PAM4	

*LNOI* lithium niobate on insulator, *N/A* not available.

^a^Operating wavelength: 1.3 µm.

### Discussion

The power dissipation of the modulator driver accounts for the great portion of the overall energy consumption of the transceivers in the datacentres. In the experiments, with the device length of 8 mm, up to 200 Gbit s^−1^ signalling is performed using the fabricated SPH modulator with a peak-to-peak drive voltage of around 1.3*V*_pp_, which is compatible with CMOS voltage levels. The corresponding electrical energy dissipation of the modulator is 42 fJ  bit^−1^ (see “Methods”). Further optimization of the loss of the microwave strip lines and the improvement of the poling efficiency could further improve the EO efficiency of the modulator, thus reducing *V*_π_, lowering the device energy consumption and facilitating the integration with CMOS circuits.

To improve the energy efficiency of the datacentres, in addition to decreasing the energy consumption in the transceivers, another effective approach is to raise the ambient temperature of the datacenters, thus reducing the energy consumed in the cooling systems. In this work, our fabricated SPH modulator allows for high-speed signalling beyond 100 Gbit s^−1^ with excellent signal fidelity at an extremely high ambient temperature of 110 °C, and shows negligible power penalty even after undergoing thermal exposure up to 90 °C for 100 h, owing to the fast response, high EO activity, and ultra-high thermal reliability (*T*_g_ = 172 °C) of the deployed side-chain EO polymer. The reliable and durable SPH modulator allows for raising the ambient operating temperature in datacentres, thus improving the running cost and energy efficiency. It could also provide EO components suitable for applications in harsh environments. For example, 5G devices are usually installed in aggressive environments (uncontrolled locations), such as on streetlamps, traffic lights, rooftops, stadiums, and parking garages, where the devices are subject to high temperatures and thermal shocks. Even in such aggressive environments, our thermal-reliable EO modulator could provide an effective solution to ensure high-throughput and reliable network connections.

The successful demonstration of the high-temperature-resistant SPH modulator operating at up to 200 Gbit s^−1^ relies on a combination of effective waveguide structures and highly efficient nonlinear organic EO materials. The presented modulator is fabricated on an ultra-thin silicon strip waveguide, featuring ease of fabrication and low propagation loss^39,40^. However, these features come at the cost of millimetre dimensions. In contrast, with device architectures based on a silicon slot waveguide^34,35,52,53^ or a metal–insulator–metal plasmonic slot waveguide^23–28^, which are deployed in SOH and POH modulators, both optical and electrical fields could be tightly confined into nanoscopic dimensions with enhanced nonlinearities and concentrated electrical field, thereby reducing the π-voltage–length product in EO modulators, and shrinking the footprint to sub-millimetre^54,55^ or micrometre^23–28^ scales. The waveguide architecture of our device could be further optimized to realize a more compact footprint, which is of utmost importance in view of future ultra-dense high-speed parallelized interconnects to facilitate dense modulator arrays^56,57^ for space-division multiplexing applications, and enable advanced complex modulations on a more compact footprint for coherent communications^45,58^. Besides, the synthesized side-chain EO polymer could be overlaid on the waveguide core as a cladding material to counterbalance the core’s TO coefficient, yielding athermal and high-speed MRRs with ultra-compact footprints^49,50^. As another important aspect of the SPH device, owing to the recent advances in material synthesis and molecular design, more efficient and thermally stable organic EO materials with higher *T*_g_ (175^59^, 194^33^, and 210 °C^60^) and improved EO activities (Pockels coefficient: 390 pm V^−1^ in device at 1.55 μm^52,53^; >290 pm V^−1^ in thin films at 1.31 μm^59^) are emerging. By incorporating such advanced organic EO materials to effective device architectures, the long-term thermal stability and EO performance of SPH modulators could be substantially improved with a compact footprint in the near future.

## Methods

### Device fabrication

The SPH modulator is fabricated on a standard 4-in. silicon wafer with a 2-μm-thick oxide layer. The bottom SiO_2_ cladding is firstly prepared on the bottom electrode of 1-μm-thick aluminium from the solution of a sol–gel trimethoxysilyl derivative. After baking the spin-coated film for the sol–gel reaction at 120–140 °C for 3 h, the 3-μm-thick bottom SiO_2_ cladding is obtained. A 40-nm-thick silicon layer is deposited by using plasma-enhanced chemical vapour deposition (SAMCO, PD-220NL). An MZI structure is then patterned onto the silicon layer by electronic beam lithography (Elionix, ELS-G100), followed by inductively coupled plasma etching (SAMCO, RIE-400iP) with SF_6_ gas. The EO polymer is then spin coated by using cyclopentanone as the solvent and baked at 105 °C for 24 h under vacuum to form a 1-µm-thick slab. A 3-µm-thick sol–gel SiO_2_ layer is then spin coated onto the EO polymer as a top cladding to protect the device during the poling and modulating processes. Travelling-wave microwave strip-line gold electrodes with a length of 8 mm are then deposited onto the device by vacuum deposition and electroplating techniques. To activate the second-order nonlinear effect in polymer, a poling voltage (300–400 V) is finally applied across the electrodes around *T*_g_ of the EO polymer to align chromophores. After cooling down the device to room temperature and removing the poling voltage, the molecular orientation is frozen, thereby enabling Pockels effects for EO modulations.

### Energy consumption of the modulator

The driver electronics of the modulators consume most of the overall energy of the transceiver. The electrical energy per bit dissipated in travelling-wave EO modulator could be calculated by *W*_bit_ = (*V*_pp_/2)^2^/(*B·R*), where *V*_pp_ is the peak-to-peak voltage swing, *B* is the line rate and *R* is the driver impendence. For our device, the transmission line impedance is designed to be matched to the 50 Ω driver circuitry. Therefore, for generating 56 Gbit s^−1^ OOK signals, the driving voltage *V*_pp_ = 1.3 V results in an electrical energy per bit of 151 fJ bit^−1^, while for 100 Gbit s^−1^ OOK systems, the corresponding electrical energy per bit is about 200 fJ bit^−1^ with *V*_pp_ = 2.0 V. In the generation of 200 Gbit s^−1^ Nyquist PAM4, the energy consumption per bit is around 42 fJ bit^−1^ with *V*_pp_ = 1.3 V. Note that with dual-drive configuration, the energy consumption could be further reduced.

### Device bandwidth characterization

To confirm the broadband operation of the SPH modulator, a vector network analyser (Anritsu, MS4647B, bandwidth: 10 MHz–70 GHz) is used to characterize the frequency response of the fabricated SPH modulator by measuring the EO S_21_ response. The modulated output optical signal is detected by an O/E calibration module (Anritsu, MN4765B-0072, frequency range: 70 kHz–70 GHz). The maximum measurable upper frequency of the characterization system is limited to 70 GHz.

### High-speed operation characterization

In the experiment for generating high-speed signals at 100 Gbit s^−1^ and beyond, the setups are depicted in Figs. 2a, b and 3a. To generate OOK signals, high-speed electrical signals generated from a pattern generator (SHF, 12104A), followed by an electronic 2:1 multiplexer (SHF, 603A), are fed into the travelling-wave electrode on the device using an RF probe (Fig. 1d, FormFactor, ACP-65-GSG-150, frequency: DC – 65 GHz, operating temperature: −65–200 °C). Another RF probe is attached to the end of the electrode with a 50 Ω termination for power absorption. In the PAM4 signalling, a four-level driving electrical signal is first obtained from an AWG (Keysight, M8196A, analog bandwidth: ~32 GHz) at a 92 GSa s^−1^ sampling rate, and fed to the SPH modulator after power amplification via a linear driver (SHF, S804B, bandwidth: 90 kHz–60 GHz). In the synthesis of Nyquist PAM4 signals, another AWG (Keysight, M8194A, analog bandwidth: ~45 GHz) at a 120 GSa s^−1^ sampling rate is used for generating the driving electrical signals. The digital signal processing at the transmitter side includes upsampling, raised cosine filtering and downsampling to form the driving electric signals. To detect the synthesized OOK and PAM4 signals, the received signal is pre-amplified by an erbium-doped fibre amplifier (EDFA) before being fed to a photo-detector (Finisar, XPDV3120R, 3-dB cut-off frequency: 75 GHz). Linear feedforward equalizers are deployed for equalizations over the digitized data in performance analysis.

### High-temperature stability assessment

To systemically assess the thermal stability of the fabricated modulators, high-speed performance of the SPH is experimentally investigated when the modulator is being operated at elevated ambient temperatures and after a burn-in test at a high storage temperature. In the performance investigation at high ambient temperatures, the modulator is mounted on an optical stage with a thermoelectric-cooler element installed, to alter the operating temperature from 25 to 110 °C. In the measurement, the device is activated with optical and electrical connections when the temperature is adjusted. On the other hand, to perform the high-temperature storage test, the device is first stored in an oven maintained at a temperature of 90 °C in air without either optical or electrical connections. After the burn-in test, the device is taken out of the oven and then optically and electrically activated to verify its high-speed functionality at room temperature. The high-speed performance of the device before the burn-in test is also conducted at room temperature, providing a benchmark for evaluating the device performance in the long-term high-temperature storage test.

## Supplementary information

Supplementary Information

## Data Availability

The datasets that support the findings of this study are available on request from the corresponding authors (G.-W.L. and S.Y.).

## Code availability

The algorithms used for this study are standard, and are outlined in “Methods”. Code scripts can be provided by the corresponding authors (G.-W.L. and S.Y.) upon reasonable request.
