# Author Correction: High-temperature-resistant silicon-polymer hybrid modulator operating at up to 200 Gbit s^−1^ for energy-efficient datacentres and harsh-environment applications

**DOI:** 10.1038/s41467-020-18908-5

**Published:** 2020-10-02

**Authors:** Guo-Wei Lu, Jianxun Hong, Feng Qiu, Andrew M. Spring, Tsubasa Kashino, Juro Oshima, Masa-aki Ozawa, Hideyuki Nawata, Shiyoshi Yokoyama

**Affiliations:** 1 Institute for Materials Chemistry and Engineering, Kyushu University, 6-1 Kasuga-koen Kasuga, Fukuoka, 816-8580 Japan; 2 The University of Aizu, Fukushima, 965-8580 Japan; 3 Department of Molecular and Material Science, Kyushu University, 6-1 Kasuga-koen Kasuga, Fukuoka, 816-8580 Japan; 4 Nissan Chemical Corporation, Funabashi, 274-0069 Japan; 5 Tokai University, Kanagawa, 259-1292 Japan

**Keywords:** Fibre optics and optical communications, Optoelectronic devices and components, Polymers, Silicon photonics

Correction to: *Nature Communications* 10.1038/s41467-020-18005-7, published online 24 August 2020.

The original version of this Article contained an error in Fig. 1c. The length of the silicon core at the center of the panel was incorrectly labelled as ‘4 mm’, rather than the correct ‘4 µm’. This has been corrected in both the PDF and HTML versions of the Article.

