# Supplementary material for: High-temperature-resistant silicon-polymer hybrid modulator operating at up to 200 Gbit s−1 for energy-efficient datacentres and harsh-environment applications
Source: Nat Commun. 2020 Aug 24;11:4224. doi: 10.1038/s41467-020-18005-7 (PMC7445259; doi:10.1038/s41467-020-18005-7)
Supplement: Supplementary file 1 — Supplementary Information [file 41467_2020_18005_MOESM1_ESM.pdf]

# Supplementary Information

# High-Temperature-Resistant Silicon-Polymer Hybrid Modulator Operating at up to 200 Gbit s$^{-1}$ for Energy-Efficient Datacentres and Harsh-Environment Applications

**Lu et al.**

# Supplementary Information

# High-Temperature-Resistant Silicon-Polymer Hybrid Modulator Operating at up to 200 Gbit s$^{-1}$ for Energy-Efficient Datacentres and Harsh-Environment Applications

Guo-Wei Lu [1,2,5,*], Jianxun Hong [1], Feng Qiu [1], Andrew M. Spring [3], Tsubasa Kashino [4], Juro Oshima [4], Masa-aki Ozawa [4], Hideyuki Nawata [4] & Shiyoshi Yokoyama [1,3,**]

[1]Institute for Materials Chemistry and Engineering, Kyushu University, 6-1 Kasuga-koen Kasuga, Fukuoka 816-8580, Japan

[2]The University of Aizu, Fukushima 965-8580, Japan

[3]Department of Molecular and Material Science, Kyushu University, 6-1 Kasuga-koen Kasuga, Fukuoka 816-8580, Japan

[4]Nissan Chemical Corporation, Funabashi 274-0069, Japan

[5]Tokai University, Kanagawa 259-1292, Japan

*gordon.guoweilu@gmail.com; **s_yokoyama@cm.kyushu-u.ac.jp

**Supplementary Note 1: Modal distribution calculation.**

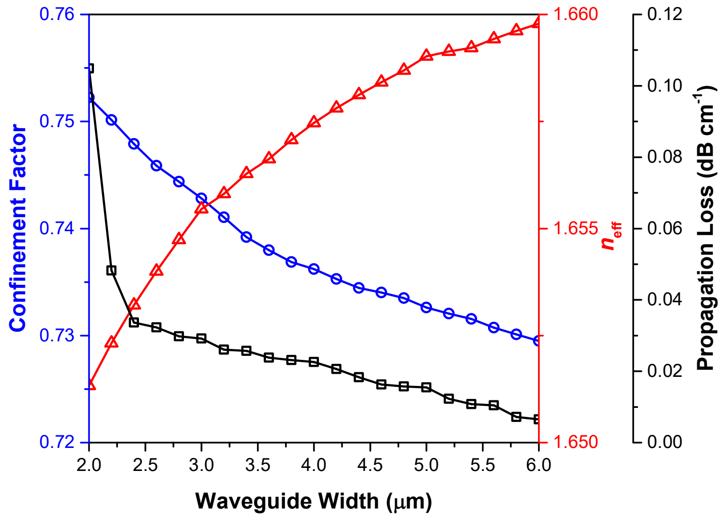


**Supplementary Figure 1 Influence of the waveguide width on the device performance.** The confinement factor (denoted as circles) in the EO polymer, the effective refractive index ($n_{\text{eff}}$, denoted as triangles) and the propagation loss (denoted as squares) are plotted as functions of the waveguide width.

The mode distribution of the fundamental TM mode is calculated by using the generalized mode solver, i.e. 3D beam propagation method (BPM), of commercial software (RSoft, BeamPROP$^{\text{TM}}$). The confinement factor ($\Gamma$) is an overlap integral factor of the light confined in the active material,

i.e. EO polymer, with the electrical field used for modulation over the total of the mode. It is defined by the following equation [1].

$$\Gamma = \frac{\iint_{\mathrm{EO}} \mathrm{Re}\{\mathbf{E}\times\mathbf{H}^*\}\cdot\mathbf{e}_z dxdy}{\iint_{\mathrm{total}} \mathrm{Re}\{\mathbf{E}\times\mathbf{H}^*\}\cdot\mathbf{e}_z dxdy} \quad (1)$$

where **E** and **H** are the electric and magnetic field vectors, respectively, and $\mathbf{e}_z$ is the unit vector in the $z$ direction. With the thickness of the silicon waveguide set to 40 nm, the corresponding confinement factor, effective refractive index and propagation loss are calculated when the waveguide width varying from 2 to 6 μm, and shown in Suppl. Fig. **1**. Note that the propagation loss is deduced from the imaginary part of the effective refractive index. With the increase of waveguide width, the confinement factor reduces, while the effective index of the mode increases. To achieve a high modulation efficiency [2], it is suggested to set the waveguide width at around 3 μm. However, considering the absorption loss and the coupling loss between the waveguide and fibre, the waveguide width is chosen to be 4 μm, leading to a confinement factor of 73.8%, which could confine the most of optical light in the EO polymer and provide a high EO efficiency for the modulation.

**Supplementary Note 2: Calculation of effective in-device EO figure of merit of the SPH.**

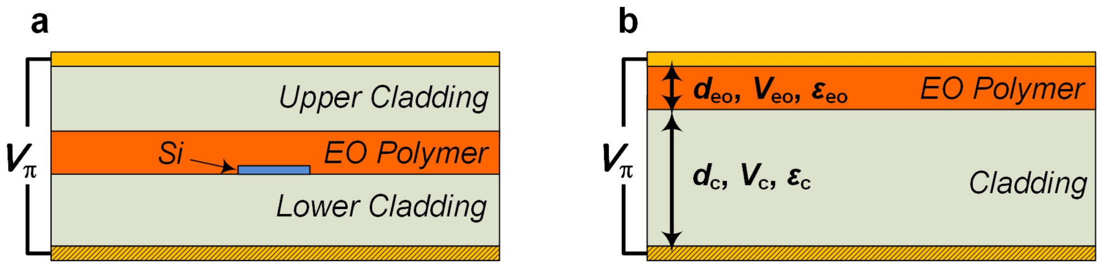


**Supplementary Figure 2 Models of SPH modulator for the calculation of the in-device EO figure of merit. a** Multilayer model. **b** Simplified two-layer model.

As shown in Suppl. Fig. **2a**, with an ultra-thin silicon core and travelling wave strip-line electrodes, the sandwich structure of the developed SPH modulator could be modelled as a multilayer dielectric structure, including upper and lower cladding layers, upper and lower electrodes, and the EO polymer layer sandwiched between them. To facilitate the analysis, by treating the upper cladding and lower cladding as one dielectric layer, it could be further simplified into a two-layer dielectric structure, shown in Suppl. Fig. **2b**. Herein, a voltage producing a phase shift of π is defined as the half-wave voltage of the modulator ($V_\pi$). According to the simplified model, the applied $V_\pi$ across the electrodes could be divided into two parts, $V_{eo}$ and $V_c$, corresponding to the distributed voltages in the EO polymer and cladding layers, respectively. Hence, we can derive the following relationships:

$$E_{eo} : E_c = \frac{1}{\varepsilon_{eo}} : \frac{1}{\varepsilon_c} \quad (2.a)$$

$$V_{\mathrm{eo}} : V_{\mathrm{c}} = \frac{d_{\mathrm{eo}}}{\varepsilon_{\mathrm{eo}}} : \frac{d_{\mathrm{c}}}{\varepsilon_{\mathrm{c}}} \tag{2.b}$$

$$V_{\mathrm{eo}} + V_{\mathrm{c}} = V_{\pi} \tag{2.c}$$

where $E_i$ and $V_i$ ($i \in$[eo, c]) are the distributed electrical field, the voltage in the EO polymer layer (denoted by subscript eo) and the cladding layer (denoted by subscript c), respectively, while $d_i$ and $\varepsilon_i$ ($i \in$[eo, c]) are the thickness and the permittivity of the EO polymer layer and cladding layer, respectively. Therefore, the distributed voltage in the EO polymer cladding could be given by

$$V_{\mathrm{eo}} = V_{\pi} \frac{d_{\mathrm{eo}}}{d_{\mathrm{eo}} + \frac{\varepsilon_{\mathrm{eo}}}{\varepsilon_{\mathrm{c}}} d_{\mathrm{c}}} = V_{\pi} \frac{d_{\mathrm{eo}}}{d_{\mathrm{eff}}} \tag{3}$$

where $d_{\mathrm{eff}}$ is the effective thickness, defined by the following equation:

$$d_{\mathrm{eff}} = d_{\mathrm{eo}} + \frac{\varepsilon_{\mathrm{eo}}}{\varepsilon_{\mathrm{c}}} d_{\mathrm{c}} \tag{4}$$

With the applied electrical field $E_{\mathrm{eo}}$ and its corresponding voltage $V_{\mathrm{eo}}$ across the EO polymer layer (thickness: $d_{\mathrm{eo}}$), the induced refractive index change ($\Delta n_{\mathrm{e}}$) and phase shift ($\Delta\theta$) could be given by the following equations:

$$\Delta n_{\mathrm{e}} = \frac{1}{2} n^3 r_{33} E_{\mathrm{eo}} \Gamma = \frac{1}{2} n^3 r_{33} \frac{V_{\mathrm{eo}}}{d_{\mathrm{eo}}} \Gamma \tag{5.a}$$

$$\Delta\theta = \frac{2\pi}{\lambda} L \cdot \Delta n_{\mathrm{e}} = \pi \tag{5.b}$$

where $L$ is the length of the phase shifter, $\lambda$ is the wavelength, $\Gamma$ is the confinement factor, $n$ is the refractive index of the active material, $r_{33}$ is the in-device EO coefficient (Pockels coefficient). Therefore, based on the above equations, the effective in-device EO figure of merit, which is defined by $n^3 r_{33}$, could be calculated from the measured $V_{\pi} \cdot L$ and $d_{\mathrm{eff}}$ by:

$$n^3 r_{33} = \frac{\lambda d_{\mathrm{eff}}}{V_{\pi} L \Gamma} \tag{6}$$

It is clear that the higher in-device EO figure of merit, the lower the $V_{\pi} \cdot L$ and hence the higher EO efficiency, indicating that it could be used to quantitatively assess the EO efficiency of the modulator. In our experiment, with the measured parameters: $\lambda$=1.55 μm, $d_{\mathrm{eo}}$=1 μm, $d_{\mathrm{c}}$=6 μm, $V_{\pi} \cdot L$=1.44 V·cm, $\Gamma$=0.738, $\varepsilon_{\mathrm{eo}} \approx 3$ and $\varepsilon_{\mathrm{c}} \approx 3$, the corresponding effective in-device EO figure of merit would be 1021 pm $V^{-1}$. Supplementary Table **1** summarizes the in-device properties of the reported state-of-the-art organic EO materials, including monolithic chromophore, binary chromophore organic glass and guest-host systems [3], deployed in silicon or metal slot waveguides. In addition, a recently-developed organic EO material consisting of cross-linked chromophores (2:1 HLD1/HLD2) shows a bulk Pockels coefficient $r_{33}$ up to ~286 pm $V^{-1}$ measured in thin films at 1.31 μm and a $T_{\mathrm{g}}$ up to 175 °C [15]. Although the in-device EO figure of merit of our fabricated material is not the highest among them, it is comparable to state-of-the-art values of the previously reported organic EO materials [6, 8, 10]. Meanwhile, it possesses the highest glass transition temperature ($T_{\mathrm{g}}$) among the

materials shown in Suppl. Table **1**, indicating excellent high-temperature thermal stability. Hence, the presented side-chain EO polymer stands out in terms of the high-temperature stability and EO efficiency in a well-balanced manner.

**Supplementary Table 1. Material in-device properties of the organic EO materials at 1.55 μm.**

| **Organic EO material** | **In-device Pockels coefficient $r_{33}$ (pm $V^{-1}$) @ 1.55 μm** | **Refractive index *n* @ 1.55 μm** | **In-device EO figure of merit (pm $V^{-1}$)** | **$T_g$ (°C)** |
|---|---|---|---|---|
| **SEO100** [4], * | 147 | 1.73 | 761 | ~150 [5] |
| **HD-BB-OH/YLD124** [6], ** | 220 | 1.77 | 1220 | 110 [7] |
| **PSLD41/YLD124** [8], * | 230 | 1.73 | 1190 | 97 [9] |
| **DLD164** [10], * | 180 | 1.88 | 1103 | 66 [11] |
| **JRD1** [12], * | 390 | 1.81 | 2313 | 82 [13] |
| **Side-chain** [This work] | 223 | 1.66 | 1021 | 172 [14] |

Note: Organic EO materials were deployed in the fabrications of *SOH and **POH.

**Supplementary Note 3: Optimization of electrode design.**

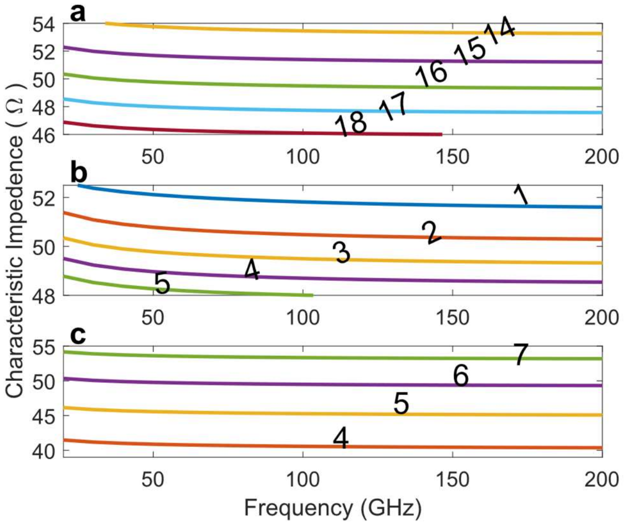


**Supplementary Figure 3 Influence of the electrode design on the characteristic impedance over a wide frequency range (20 GHz~200 GHz). a** The estimated characteristic impedance when the electrode width varies from 14 μm to 18 μm and the electrode thickness is fixed at 3 μm. **b** The estimated characteristic impendence when the electrode thickness varying from 1 μm to 5 μm while the thickness fixed at 16 μm. **c** The estimated characteristic impendence when the total thickness of the cladding layers varies from 4 μm to 7 μm while the electrode dimensions are fixed with a size of 16 μm×3 μm.

In the design of a travelling-wave EO modulator, as two crucial aspects, the microwave attenuation and the phase velocity mismatching between the microwave and light signals are critically affected by the electrode design [16, 17]. Therefore, the electrode design should be optimized for wide

bandwidth and high-speed operations. Particularly, the dimension of the electrodes should be optimized to make the characteristic impendence close to 50 Ω over a wide frequency range, thus avoiding the reduction of electrode RF bandwidth due to impedance mismatch induced reflection. Herein, a numerical simulation is conducted using a finite element method (FEM, CST Studio Suite) to investigate the influence of electrode parameters on the characteristic impedance across high frequencies. Given that the cladding thickness is 6 μm, Suppl. Fig. **3a** depicts the estimated characteristic impedance over a frequency range between 20 GHz and 200 GHz with the electrode width varying from 14 μm to 18 μm and the electrode thickness being fixed at 3 μm. Supplementary Figure **3b** shows the estimated characteristic impedance across the same frequency range when the electrode thickness varies from 1 μm to 5 μm while the electrode width is fixed at 16 μm. It is clear that when the thickness and the width of the electrode are chosen as 3 μm and 16 μm, respectively, the characteristic impedance is close to 50 Ω over a large frequency range. Furthermore, the characteristic impedances are estimated at different frequencies when the cladding thickness is adjusted and the electrode dimensions are fixed with a thickness of 3 μm and a width of 16 μm. Here we assume the device has symmetric cladding layers, i.e. the upper and lower cladding layers have the same thickness. As shown in Suppl. Fig. **3c**, with a total cladding thickness of 6 μm, the characteristic impedance is maintained close to 50 Ω over a wide frequency range. Therefore, the electrode dimensions are optimized with a 16 μm width and a 3 μm thickness, while the optimal total thickness of the cladding layer is 6 μm. The optimized electrode design could ensure a broadband impendence match and effectively avoid bandwidth degradation.

**Supplementary Note 4: Thermal reliability assessment at 100 Gbit s$^{-1}$ and beyond.**

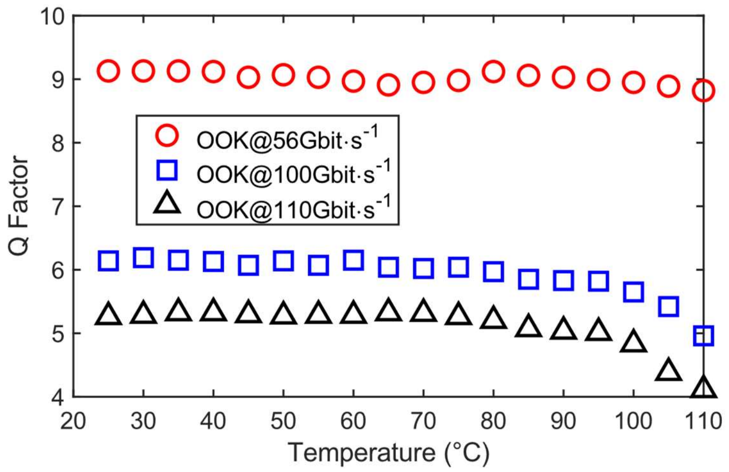


**Supplementary Figure 4 Measured *Q* factors of OOK signals at bit rates of 56 Gbit s$^{-1}$, 100 Gbit s$^{-1}$, and 110 Gbit s$^{-1}$ versus the operating ambient temperature (25~110 °C).**

Supplementary Figure **4** illustrates the measured *Q* factors (in linear scale) of the synthesized OOK signals from the device when the operating ambient temperature varies from 25 °C to 110 °C.

**Supplementary Note 5: Propagation loss characterization**

The fiber-to-fiber insertion loss of the fabricated SPH modulator is found to be ~10 dB, which is attributed to the scattering losses in the active phase shifter, the losses in passive components such as Y-junctions (~1.4 dB in total), and the edge coupling losses of lensed fibers (~3 dB per facet).

Thus, the on-chip optical loss is estimated to be approximately 2.6 dB, resulting in a waveguide propagation loss of 0.22 dB $mm^{-1}$ by considering the total length of the device of 12 mm. It is also confirmed by measuring the propagation loss of a straight reference waveguide. It is expected the loss could be further reduced by optimizing the waveguide structure and fabrication process [18-21].

## Supplementary References


[1] Robinson, J. T., Preston, K., Painter, O. & Lipson M. First-principle derivation of gain in high-index-contrast waveguides. *Opt. Express* **16**, 16659–16669 (2008).

[2] Himmelhuber, R. et al. A silicon-polymer hybrid modulator-design, simulation and proof of principle. *J. Lightwave Technol.* **31**, 4067–4072 (2013).

[3] Heni, W. et al. Nonlinearities of organic electro-optic materials in nanoscale slots and implications for the optimum modulator design. *Opt. Express* **25**, 2627–2653 (2017).

[4] Wolf, S. et al. Silicon-organic hybrid (SOH) Mach-Zehnder modulators for 100 Gbit/s on-off keying. *Sci Rep* **8**, 2598 (2018) doi:10.1038/s41598-017-19061-8.

[5] Jouane, Y. et al. Unprecedented highest electro-optic coefficient of 226 pm/V for electro-optic polymer/TiO2 multilayer slot waveguide modulators. *Opt. Express* **22**, 27725–27732 (2014).

[6] Haffner, C. et al. Harnessing nonlinearities near material absorption resonances for reducing losses in plasmonic modulators. *Opt. Mater. Express* **7**, 2168–2181 (2017).

[7] Elder, D. L. et al. Effect of rigid bridge-protection units, quadrupolar interactions, and blending in organic electro-optic chromophores. *Chem. Mater.* **29**, 6457–6471 (2017).

[8] Palmer, R. et al. High-speed, low drive-voltage silicon-organic hybrid modulator based on a binary-chromophore electro-optic material. *J. Lightwave Technol.* **32**, 2726–2734 (2014).

[9] Pereverzev, Y. V. et al. Guest−host cooperativity in organic materials greatly enhances the nonlinear optical response. *J. Phys. Chem. C* **112**, 4355–4363 (2008).

[10] Koeber, S. et al. Femtojoule electro-optic modulation using a silicon–organic hybrid device. *Light Sci Appl* **4**, e255 (2015).

[11] Elder, D. L., Benight, S. J., Song, J., Robinson, B. H. & Dalton, L. R. Matrix-assisted poling of monolithic bridge-disubstituted organic NLO chromophores. *Chem. Mater.* **26**, 872–874 (2014).

[12] Kieninger, C. et al. Ultra-high electro-optic activity demonstrated in a silicon-organic hybrid modulator. *Optica* **5**, 739–748 (2018).

[13] Jin, W. et al. Structure–function relationship exploration for enhanced thermal stability and electro-optic activity in monolithic organic NLO chromophores. *J. Mater. Chem. C* **4**, 3119–3124 (2016).

[14] Sato, H. et al. Low driving voltage Mach-Zehnder interference modulator constructed from an electro-optic polymer on ultra-thin silicon with a broadband operation. *Opt. Express* **25**, 768–775 (2017).

[15] Xu, H. et al. Ultrahigh electro-optic coefficients, high index of refraction, and long-term stability from Diels-Alder cross-linkable binary molecular glasses. *Chem. Mater.* **32**, 1408–1421 (2020).

[16] Hui, K. W., Chiang, K. S., Wu, B. & Zhang, Z. H. Electrode optimization for high-speed traveling-wave integrated optic modulators. *J. Lightwave Technol.* **16**, 232–238 (1998).

[17] Zang, D. Y., Meng, X., Lin W. & Bechtel, J. H. Optimization of microstrip electrode design for high-speed polymer electro-optic modulator. In *Proc. SPIE 4987, Integrated Optics: Devices, Materials, and Technologies VII.* 188–196 (2003).

[18] Wang, X. et al. Continuously tunable ultra-thin silicon waveguide optical delay line. *Optica* **4**, 507–515 (2017).

[19] Zou, Z., Zhou, L., Li, X. & Chen, J. 60-nm-thick basic photonic components and Bragg gratings on the silicon-on-insulator platform. *Opt. Express* **23**, 20784–20795 (2015).

[20] Li, C. & Dai, D. Low-loss and low-crosstalk multi-channel mode (de)multiplexer with ultrathin silicon waveguides. *Opt. Express* **42**, 2370–2373 (2017).

[21] Cardenas, J. et al. Low loss etchless silicon photonic waveguide. *Opt. Express* **17**, 4752–4757 (2009).
